# Synergistic Effects of BMP9 and miR-548d-5p on Promoting Osteogenic Differentiation of Mesenchymal Stem Cells

**DOI:** 10.1155/2015/309747

**Published:** 2015-11-01

**Authors:** Wei Zhang, LiCheng Zhang, Yan Zhou, XinRan Ji, JianHeng Liu, DaoHong Liu, Peng Yin, Ye Peng, Ming Hao, LiHai Zhang, PeiFu Tang

**Affiliations:** ^1^Department of Orthopaedics, General Hospital of Chinese PLA, No. 28 Fuxing Road, Haidian District, Beijing 100853, China; ^2^Medical Department, Affiliated Hospital of Chinese PLA General Hospital, Beijing 100048, China

## Abstract

Various stimulators have been reported to promote MSC osteogenic differentiation via different pathways such as bone morphogenetic protein 9 (BMP9) through influencing COX-2 and miR-548d-5p through targeting peroxisome proliferator-activated receptor-*γ* (PPAR*γ*). Whether synergistic effects between BMP9 and miR-548d-5p existed in promoting osteogenesis from MSCs was unclear. In the study, the potential synergistic effects of BMP9 and miR-548d-5p on human MSC differentiation were investigated. Osteogenic differentiation of MSCs treated with BMP9 or miR-548d-5p was detected with multimodality of methods. The results demonstrated that BMP9 and miR-548d-5p significantly influenced COX-2 and PPAR*γ*, respectively. BMP9 also influenced the expression of PPAR*γ*, but no significant effect of miR-548d-5p on COX-2 was observed. When BMP9 and miR-548d-5p were combined, more potent effects on both COX-2 and PPAR*γ* were observed than BMP9 or miR-548d-5p alone. Consistently, osteogenic analysis at different timepoints demonstrated that osteogenic genes, ALP activity, calcium deposition, OPN protein, and matrix mineralization were remarkably upregulated by BMP9/miR-548d-5p compared with BMP9 or miR-548d-5p alone, indicating the synergetic effects of BMP9 and miR-548d-5p on osteogenic differentiation of MSCs. Our study demonstrated that regulating different osteogenic regulators may be an effective strategy to promote bone tissue regeneration for bone defects.

## 1. Introduction

Mesenchymal stem cells (MSCs) are multipotent cells that are able to differentiate into a series type of cells, such as osteoblasts, adipocytes, chondrocytes, or myocytes [1–4]. MSCs can be obtained from multiple sources, including adipose, periosteum, and bone marrow [5–8]. The fine-tuned balance between osteogenesis and adipogenesis of MSCs is important to a variety of human diseases, for instance, osteonecrosis, osteoporosis, and age-related bone loss [9, 10].

Bone morphogenetic proteins (BMPs), belonging to TGF-*β* superfamily, play a critical role in bone development and osteogenic differentiation [11, 12]. Among members of BMPs, BMP9 was reported to be one of the most potent BMPs to stimulate osteogenic differentiation of MSCs both* in vivo* and* in vitro* [13–16]. For BMP9 induced osteogenic differentiation, a few important downstream targets were identified, including COX-2 [17, 18], Hey1 [19], and Creld2 [20]. COX-2 belongs to cyclooxygenase (COX) family, which produces prostaglandins (PGs) with arachidonic acid [18]. Among identified 3 types of COX, namely, COX-1, COX-2, and COX-3, COX-2 was demonstrated to be the only one that plays an important role in bone metabolism [21, 22]. Previous studies indicated that COX-2 can also promote BMP9 induced osteogenic differentiation through BMP9/Smads signal pathways [17, 18].

Peroxisome proliferator-activated receptor gamma (PPAR*γ*), as an important cell-fate-defining factor, has been demonstrated to be able to promote adipogenesis [23, 24]. A novel strategy has been addressed that suppression of PPAR*γ* could improve the bone regeneration [25] and MSCs osteogenic differentiation [26]. One effective method of downregulating PPAR*γ* was to introduce miRNAs. Previous study found that miR-548d-5p was able to downregulate PPAR*γ* by targeting its mRNA 3′-UTR [27] and thus enhanced MSC osteogenic potential and blocked its adipogenesis.

The different mechanisms by BMP9 and miR-548d-5p in promoting MSC osteogenic differentiation made us hypothesize that simultaneously regulating different osteogenic regulators may produce more potent osteogenesis from MSCs, which, however, was not demonstrated. Therefore, we designed a series of experiments in the study to assess the effects of BMP9 and miR-548d-5p on osteogenic differentiation of human adipose-derived MSCs simultaneously.

## 2. Materials and Methods

### 2.1. Ethics Statement

To obtain adipose-derived MSCs, raw human adipose tissue collection and cell harvests were approved by the Chinese People's Liberation Army General Hospital's Protection of Human Subjects Committee. Subjects have received an explanation about the scope of the study and signed an informed consent statement before donation in the study.

### 2.2. Isolation of Adipose-Derived Mesenchymal Stems Cells (MSCs)

The human adipose-derived MSCs were isolated from raw human lipoaspirates and cultured as the previous report [28, 29]. Briefly, clear lipoaspirates were firstly obtained through washing with phosphate buffer saline (PBS). After removing contaminating debris and red blood cells, 45 min digestion (0.1% collagenase I from Sigma in serum-free *α*MEM) was conducted. Then, equal volume of *α*MEM (10% fetal bovine serum from FBS, Gibco) was supplemented to inhibit the trypsin. The mixture was sieved through 80 *μ*m mesh. The cell part was allowed to plate on tissue culture dishes and incubated at 37°C and 5% CO_2_. The adherent cells were employed as ADSCs and passages of MSCs were limited to 5.

### 2.3. miR-548d-5p Transfection

The miR-548d-5p agomir (GMR-miR microRNA-548d-5p agomir, Shanghai GenePharma Co. Ltd.) was designed and synthesized. When cells confluence achieved ~50%, transfection of miR-548d-5p was performed using Lipofectamine RNAiMAX Transfection Reagent (Life Technologies) according to the manufacturer's instructions. The final concentration of miR-548d-5p agomir was set at 50 nM.

### 2.4. Recombinant Adenovirus Construction Expressing Bone Morphogenetic Protein 9 (BMP9)

Recombinant BMP9 adenoviruses were obtained through AdEasy technology as described previously [30, 31]. The coding regions of BMP9 were amplified by PCR. The PCR products were cloned into an adenoviral shuttle vector. The vector was then applied to generate recombinant adenoviruses in HEK-293 cells. The prepared adenoviruses were designated as AdBMP9.

### 2.5. Quantitative RT-PCR

For cultured MSCs cells, total RNA was isolated from at least 1 × 10^5^ cells by Total RNA Kit (OMEGA, Norcross, GA, USA), according to the manufacturer's protocol. After quantification, RNA samples with A260/A280 nm ratio more than 1.8 were retained and applied in the following experiments. 2 *μ*g of total RNA was reversely transcribed into 1st-strand cDNA with PrimeScript RT reagent kit (Takara, Shiga, Japan). The obtained cDNA was then subjected to qRT-PCR analysis and the sequence of primers for PCR was as follows: GAPDH, 5′-ACC ACA GTC CAT GCC ATC AC-3′ (sense) and 5′-TCC ACC ACC CTG TTG CTG TA-3′ (antisense); COX-2, 5′-GTC ACA AGA TGG CAA AAT GCT G-3′ (sense) and 5′-TAA GAT AAC ACT GCA GTG GCT C-3′ (antisense); PPAR*γ*, 5′-ACT CTG GGA GAT TCT CCT ATT-3′ (sense) and 5′-CTC CAT AGT GAA ATC CAG AAG-3′ (antisense); Runx2, 5′-GCA CCG ACA GCC CCA ACT T-3′ (sense) and 5′-CCA CGG GCA GGG TCT TGT T-3′ (antisense); OCN, 5′-TGA GGA CCC TCT CTC TGC TC-3′ (sense) and 5′- GGG CTC CAA GTC CAT TGT T-3′ (antisense); OPN, 5′-ATC TGA GTC CTT CAC TG-3′ (sense) and 5′-GGG ATA CTG TTC ATC AGA AA-3′ (antisense); Col I, 5′-TGT TCG TGG TTC TCA GGG TAG-3′ (sense) and 5′-TTG TCG TAG CAG GGT TCT TTC-3′ (antisense); BSP, 5′-ATA GGC AAC GAG TAC AAC AC-3′ (sense) and 5′-GTA TCC AGA TGC AAA GAC AG-3′ (antisense). In addition, PCR products were analyzed with agarose gel electrophoresis.

### 2.6. Western Blotting Analysis

MSCs were lysed in Laemmli Sample Buffer (Bio-Rad). Proteins were collected by centrifugation and concentrations were determined by BCA Protein Assay Kit (Thermo Scientific). Proteins were loaded on sodium dodecyl sulfate polyacrylamide gel for electrophoresis (SDS-PAGE). After proteins were transferred to nitrocellulose membranes, primary antibodies against PPAR*γ* (Abcam) and COX-2 (Abcam) were incubated overnight at 4°C. Then, corresponding secondary antibodies were incubated for 1 h at room temperature. GAPDH was used as internal standard.

### 2.7. Flow Cytometry Analysis

The MSCs of 10 days culture were rinsed with PBS and fixed with 4% paraformaldehyde. Subsequently, after treatment with 0.2% Triton X-100, 5% bovine serum albumin (BSA) was utilized for terminating the reaction. The cells were incubated with primary antibody specified for osteopontin (OPN) overnight at 4°C and then corresponding secondary antibodies conjugated FITC (Abcam) for 1 h at room temperature. Fluorescence-activated cell sorting caliber flow cytometry system (FACS Caliber BD Flow Cytometer) was used for data analysis.

### 2.8. Alkaline Phosphatase (ALP) Activity Assay

After osteogenic induction culturing for 3, 7, and 10 days, cells were rinsed twice and treated with 15 s sonication in 2 mL buffer (50 mM pH 7.2 Tris-HCl, 0.1% Triton X-100, and 2 mM MgCl_2_). The measurement of ALP activity was performed with a previous method with minor modification using a commercial ALP Detection Kit (Nanjing Jiancheng Bioengineering Ltd., Nanjing, China) [32]. The ALP data were described as nmol/15 min/mg protein.

### 2.9. Osteocalcin Content Analysis

The culture mediums at osteogenic induction culturing for 3, 7, and 10 days were gathered. The detection of the concentration of osteocalcin was conducted through enzyme immunoassay (ELISA) using an osteocalcin kit as instructed (Immunodiagnostic Systems Ltd., Boldon, UK) [32].

### 2.10. Matrix Mineralization Assay

Matrix mineralization was performed by alizarin red S staining, as described previously [33]. At 2 and 3 w after treatment, cells were fixed with 10% formaldehyde for 10 min, following incubation of 40 mM alizarin red S (Sigma) at 37°C for 1 h. After careful washing, the staining cultures were recorded by a light field microscope.

### 2.11. Measurement of Calcium Deposition Level

At the 2 w for osteogenic induction culture, the cells were washed twice with PBS buffer and then supplemented with 1 mL of 1 M HCl, following cell incubation with gentle shaking overnight. The free [Ca^2+^] in the well was analyzed by the O-cresolphthalein complex method with Calcium Colorimetric Assay Kit (BioVision) [34].

### 2.12. Statistical Analysis

Values were expressed as mean ± standard deviation (SD). *P* values < 0.05 were considered as statistically significant. Analysis between the groups was performed by using 1-way ANOVAs followed by Tukey's* post hoc* test for multiple pairwise examinations.

## 3. Results

### 3.1. The Effects of BMP9 and miR-548d-5p on COX-2 and PPAR*γ*

**Figure 1:**
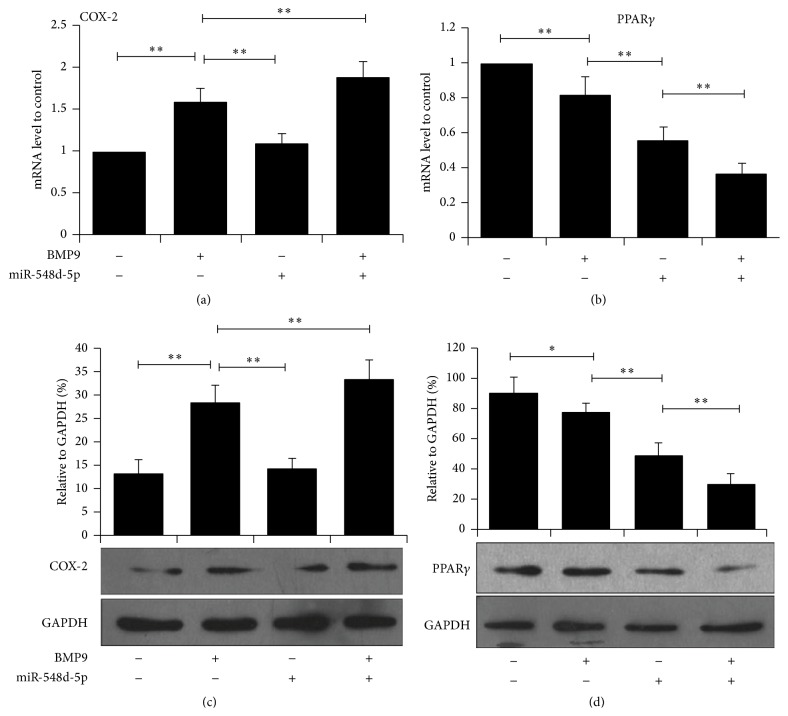
Combination of BMP9 and miR-548d-5p upregulates COX-2 expression and downregulates PPAR*γ*. (a, b) RT-PCR shows the synergetic effects of BMP9 and miR-548d-5p on COX-2 and PPAR*γ* in MSCs (^*∗∗*^ *P* < 0.01). (c, d) Western blot assay shows the effects of BMP9 and miR-548d-5p on COX-2 and PPAR*γ* in MSCs (^*∗∗*^ *P* < 0.01).

Adipose-derived MSCs were transfected with BMP9 and/or miR-548d-5p. Both gene expression and protein expression were analyzed by RT-PCR and western blotting, respectively. We first tested the effects of BMP9 and miR-548d-5p on COX-2 expression. Compared with the control, BMP9 infection could dominantly enhance the expression of COX-2 in accordance with previous study [18], while miR-548d-5p alone had negligible effects on COX-2. When BMP9 and miR-548d-5p were employed simultaneously, COX-2 was further enhanced compared with BMP9 alone (Figure 1(a), *P* < 0.01). Then, the expression level of PPAR*γ* was examined. As shown in Figure 1(b), both BMP9 and miR-548d-5p inhibited the expression of PPAR*γ*, and more potent inhibition effect was observed by miR-548d-5p. When BMP9 and miR-548d-5p were combined, the expression of PPAR*γ* was significantly lower than BMP9 and miR-548d-5p alone. These results indicated that BMP9 and miR-548d-5p may play synergetic roles in regulating osteogenic regulators, COX-2 and PPAR*γ*. The effects of BMP9 and miR-548d-5p on COX-2 and PPAR*γ* expression were further verified by western blotting. As shown in Figures 1(c) and 1(d), western blotting from protein levels achieved consistent results with RT-PCR from gene levels.

### 3.2. Expression of Osteogenic Genes

**Figure 2:**
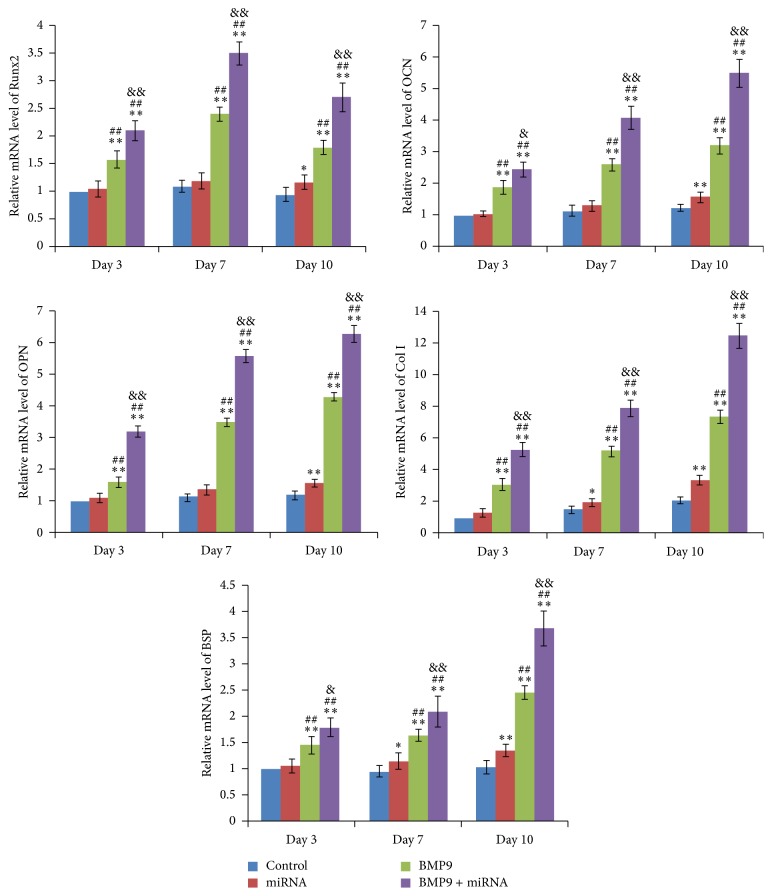
Combination of BMP9 and miR-548d-5p upregulates osteogenic differentiation-related factors in MSCs. RT-PCR demonstrated that Runx2, OPN, OCN, Col I, and BSP were significantly unregulated in BMP9 and miR-548d-5p treated MSCs at day 3, day 7, and day 10, respectively (^*∗∗*^ *P* < 0.01 versus control; ^##^ *P* < 0.01 versus miR-548d-5p; ^&&^ *P* < 0.01 versus BMP9).

In the different time points of osteogenic differentiation, osteogenic gene markers were analyzed by RT-PCR. The mRNA expression levels of Runx2, OCN, OPN, Col I, and BSP were analyzed at days 3, 7, and 10, respectively. As showed in Figure 2, miR-548d-5p infection alone could trigger a time-dependent minor increase in transcript expression of all these osteogenic markers. The transfection by BMP9 enhanced those mRNA expressions predominantly with differentiation time. Compared with BMP9 and miR-548d-5p alone, combination of BMP9 and miR-548d-5p treatment significantly enhanced the expression of all the five markers. These results suggested that BMP9 stimulation was a potent osteogenic factor, which directly promoted MSC osteogenic differentiation. Interestingly, we could presume that downregulation of PPAR*γ* may indirectly reinforce the osteogenic potential of MSCs rather than directly promote their osteogenic differentiation. Thus, when cultured under osteogenic conditions, the potent effects of PPAR*γ* downregulation on osteogenic differentiation could be manifested by synergism with osteogenic stimulators.

### 3.3. ALP Activity and Osteocalcin Content

**Figure 3:**
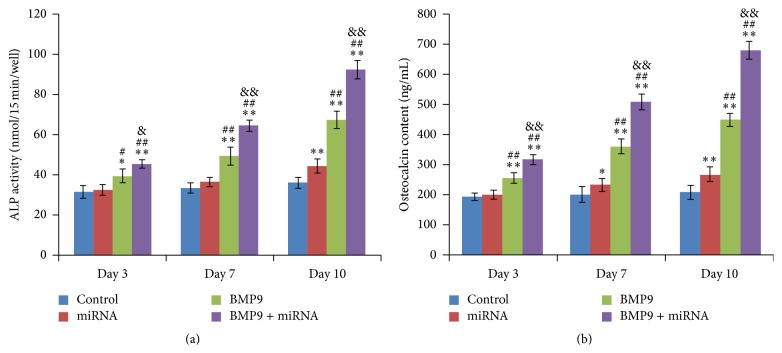
Combination of BMP9 and miR-548d-5p promotes ALP activities and augments OCN content in MSCs. (a) The synergetic effects of BMP9 and miR-548d-5p on ALP activities in MSCs at day 3, day 7, and day 10, respectively (^*∗∗*^ *P* < 0.01 versus control; ^##^ *P* < 0.01 versus miR-548d-5p; ^&&^ *P* < 0.01 versus BMP9). (b) The synergic effects of BMP9 and miR-548d-5p on OCN content in MSCs at day 3, day 7, and day 10, respectively (^*∗∗*^ *P* < 0.01 versus control; ^##^ *P* < 0.01 versus miR-548d-5p; ^&&^ *P* < 0.01 versus BMP9).

The activity of intracellular ALP was also investigated at days 3, 7, and 10. The activity of ALP in the treatment groups significantly increases with time. Both the solo treatment by BMP9 and combination of BMP9 and miR-548d-5p could enhance the activity largely, while miR-548d-5p infection alone could stimulate a relatively slight increase. Of the three ways of treating MSCs, the cotreatment showed a clear superiority compared to the other two, as shown in Figure 3(a). Then, the osteocalcin secretion was also examined at the same timepoint with ALP analysis (days 3, 7, and 10). As for osteocalcin contents, similar results were also obtained (Figure 3(b)). These findings further supported the assumption that BMP9 acted as a potent direct promoter of MSCs osteogenic differentiation which could be further strengthened by indirect enhancement of miR-548d-5p through inhibiting PPAR*γ*.

### 3.4. Flow Cytometry of Osteopontin (OPN)

**Figure 4:**
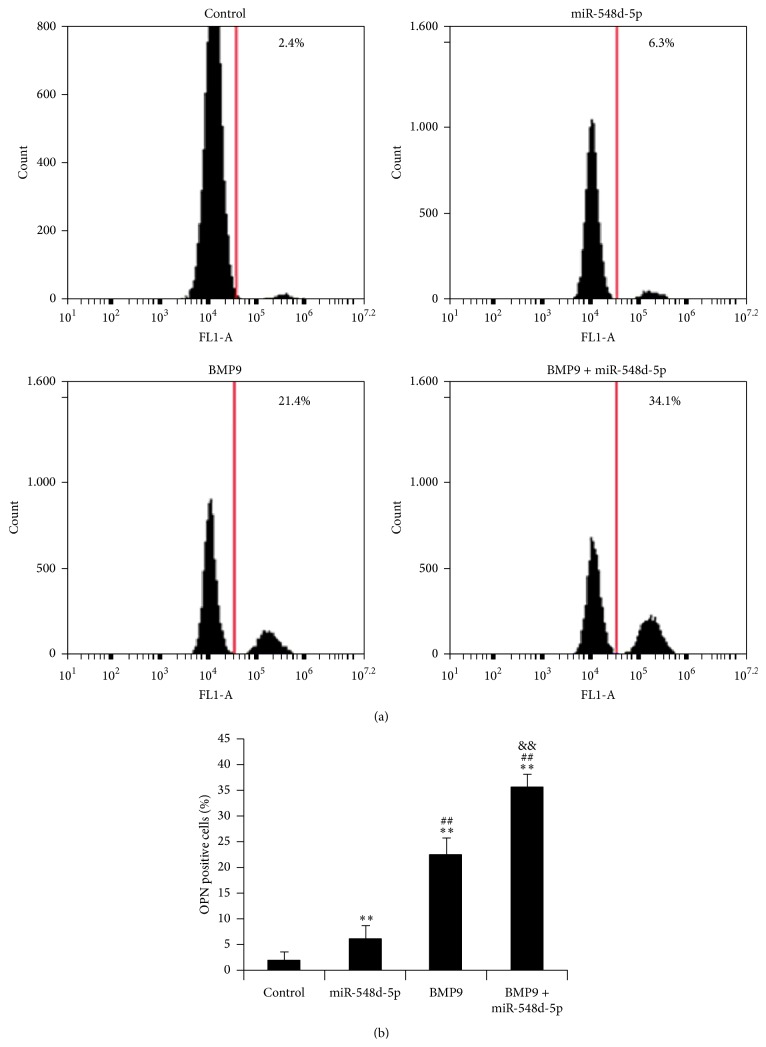
Fluorescence-activated cell sorting (FACS) analysis performed on the MSCs cultured for 10 days. OPN immunostained MSCs were measured by a flow cytometry analysis system (*y*-axis: the cell number; *x*-axis: fluorescence intensity). The data shows the synergetic effects of BMP9 and miR-548d-5p on OPN expression of MSCs at day 10 (^*∗∗*^ *P* < 0.01 versus control; ^##^ *P* < 0.01 versus miR-548d-5p; ^&&^ *P* < 0.01 versus BMP9).

The combined impact of BMP9 and miR-548d-5p on protein expression of OPN, the late osteogenic marker, has then been investigated. According to FACS results of Figure 4, the expression of OPN was augmented about 2.5-fold by a separate administration of miR-548d-5p. However, a 9-fold increase of OPN expression was observed, after introduction of BMP9 alone. Moreover, the combination of BMP9 and miR-548d-5p could further enhance the expression of OPN and cause an over 14-fold augmentation of expression. The higher OPN overexpression induced by the combination of BMP9 and miR-548d-5p compared to simple summation of BMP9 and miR-548d-5p similarly suggested potential synergism of BMP9 and PPAR*γ* inhibitor.

### 3.5. Matrix Mineralization and Calcium Deposition of MSCs

**Figure 5:**
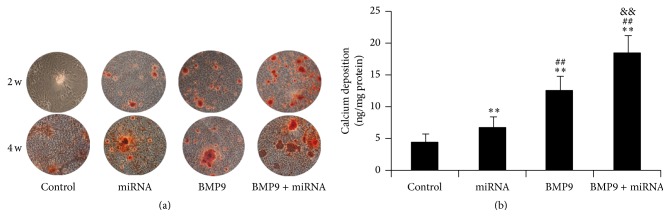
Combination of BMP9 and miR-548d-5p enhances matrix mineralization and calcium deposition in MSCs. (a) Alizarin red S staining at 2 and 3 w shows the synergetic effects of BMP9 and miR-548d-5p on matrix mineralization; (b) the synergetic effects of BMP9 and miR-548d-5p on calcium deposition in MSCs cultured for 14 days (^*∗∗*^ *P* < 0.01 versus control; ^##^ *P* < 0.01 versus miR-548d-5p; ^&&^ *P* < 0.01 versus BMP9).

Finally, we determined the synergistic effects of BMP9 and miR-548d-5p on matrix mineralization and calcium deposition. We found that matrix mineralization assessed by alizarin red staining was remarkably promoted by the combination of BMP and miR-548d-5p, compared with their separate treatment (Figure 5(a)). Additionally, quantitative analysis of calcium deposition similarly showed that BMP9 induced apparently osteogenic calcium secretion compared with treatment by PPAR*γ* inhibiting, and a more enhancement of calcium secretion was achieved when BMP9 accompanied with PPAR*γ* inhibitor was introduced (Figure 5(b)). Taken together, the results of late stage in MSCs further suggested that inhibiting PPAR*γ* could enforce BMP9 induced osteogenic differentiation.

## 4. Discussion

Osteogenic differentiation of stem cells is a highly orchestrated process in which a series of factors are involved. BMPs [35–37], IGF [38], PI3K/Akt [17], miRNA [27, 39], drugs [33], and even microenvironment [40, 41] are able to regulate osteogenic differentiation. Albeit various, they could be mainly concluded into two pathways. The first one is to tempt stem cells to the way to osteogenic differentiation, such as BMP9. BMP9 belongs to the family of BMPs, which play an important role in both bone metabolism and tumor formation [11, 12]. Of all BMPs members, BMP9 was considered to be the most potential to enhance osteogenic differentiation [13–16]. As recent reports, the regulative function of BMP9 was fulfilled by possibly forming a loop structure with COX-2 and then orchestrating the BMP9/Smad signal pathway [18]. The other is to block stem cells to other ways of differentiation, for example, miR-548d-5p. miRNAs have been demonstrated to be potent to regulate stem cells differentiation [27, 39]. Of them, miR-548d-5p was found to be able to inhibit adipogenesis of MSCs by binding to the mRNA of PPAR*γ* [27]. Thus, more cells were defined to osteogenesis. However, no such study as efforts from both two directions is reported. Therefore, we firstly choose BMP9 representing the tempting way and miR-548-5p representing the blocking way to explore possible synergetic effects on MSCs osteogenic differentiation.

COX-2 plays a critical role in bone metabolism and has also been proved to be essential for BMP9 induced osteogenic differentiation [17, 18]. In current study, we found that the combination of miR-548d-5p and BMP9 could further promote the gene and protein level expressions of COX-2 compared to BMP9 alone, while the separate miR-548d-5p treatment caused negligible impacts on it. This indicated that miR-548d-5p could possibly enhance the BMP9 induced upregulation of COX-2. In addition, the expression of PPAR*γ*, one of most important factors to promote adipogenic differentiation in MSCs, was examined. PPAR*γ* can be exclusively mutated by miR-548d-5p through targeting 3′-UTR of PPAR*γ*. We found that BMP9 could also significantly, albeit not remarkably, reduce the expression of PPAR*γ*. The downregulation of PPAR*γ* by BMP9 possibly contributed to feedback effects associated with BMP9 induced osteogenic differentiation. And the combination of BMP9 and miR-548d-5p showed the strongest inhibiting effects on PPAR*γ*.

We next found that the combination of BMP9 and miR-548d-5p could also improve early and late osteogenic differentiation markers of MSCs, as well as matrix mineralization, predominantly. In conformity with the results of COX-2, the expressions of BMP9 induced osteogenic differentiation downstream markers [42], including Runx2, OCN, OPN, Col I, and BSP, were remarkably upregulated by cotreatment of BMP9 and miR-548d-5p, while they were slightly enhanced in presence of miR-548d-5p alone. These findings further supported the hypothesis that miR-548d-5p promoted BMP9 induced osteogenic differentiation through silencing the expression of PPAR*γ*. Moreover, similar results of ALP activity, calcium deposition, and matrix mineralization also corresponded to the hypothesis. It is needed to state that the terminal differentiation of the differentiated MSCs may last for longer time than we observed in the study. However, it was observed during our experiment that when differentiation was proceeded for 3 w or longer time, the state of the differentiated MSCs would become much worse than that at 2 w due to the long-time culture, and some cells and calcified tubercles would easily fall off culture plate (this could be seen in Figure 5(a), alizarin red S staining at 3 w). This would result in large deviation for calcium deposition quantification. That was why we chose 2 w as the timepoint to quantify calcium deposition in the study (Figure 5(b)).

As mentioned above, the results of BMP9 induced osteogenic differentiation were in conformity with previous studies [11, 17, 18, 33]. BMP9 could apparently upregulate a series of osteogenesis factors in both early and late stage of MSCs, due to its direct involvement of several osteogenic differentiation pathways. However, taking the results of slight osteogenic enhancement of MSCs and large osteogenic improvement of BMP9 induced MSCs by PPAR*γ* inhibiting together, we proposed the hypothesis that PPAR*γ* inhibiting can promote osteogenic differentiation of those osteogenic induced MSCs but cannot stimulate those uncertain MSCs to osteogenic differentiation. PPAR*γ* is one of the most important factors in balancing adipogenesis and osteogenesis in MSCs. Overexpression of PPAR*γ* positively regulates adipogenesis but negatively regulates osteogenesis. However, downregulation of PPAR*γ* alone is probably insufficient to effectively trigger osteogenesis-related pathways. Moreover, only under proper beneficial osteogenic conditions and osteogenesis-related pathways activated, PPAR*γ* downregulation would ensure osteogenic differentiation thoroughly and successfully to some extent and thus largely promote MSCs osteogenic differentiation. Additionally, there were conflicts in whether PPAR*γ* silencing was able to promote MSCs osteogenesis [26, 43]. Our assumption may serve to enlighten possible understanding of the contention. Although the* in vitro* data well confirmed the synergic effects of BMP9 and miR-548d-5p on promoting osteogenic differentiation of MSCs, we still cannot be sure that it would work well* in vivo* and this should be a future endeavor.

In conclusion, our study demonstrates that the combination of BMP9 and miR-548d-5p can promote osteogenic differentiation of MSCs. This synergic effect is probably due to the improvement of BMP9 induced osteogenic differentiation by mutated PPAR*γ* with miR-548d-5p. And the subsequent upregulation of Runx2, OPN, OCN, Col I, and BSP was also enhanced. Our findings may not only shed light on the possible mechanism of synergic effect on osteogenic differentiation from two different directions but also provide an effective strategy to promote skeletal tissue regeneration of osteonecrosis.
